# First cases of suspected sexual transmission of *Trichophyton mentagrophytes* genotypes VII and VIII in Orange County, California, USA

**DOI:** 10.1128/asmcr.00011-26

**Published:** 2026-06-04

**Authors:** Shannen Guarina, Amirhossein Darvishzadeh, Cassiana Bittencourt, Rosa M. Andrade

**Affiliations:** 1 School of Medicine, University of California Irvine 8788, Irvine, California, USA; 2 Department of Internal Medicine, Division of Infectious Diseases, University of California Irvine 8788, Irvine, California, USA; 3 Department of Pathology, University of California Irvine 8788, Irvine, California, USA; 4 Department of Internal Medicine, Division of Infectious Diseases, Microbiology and Molecular Genetics, Program in Medical Education for the Latino Community (PRIME-LC), School of Medicine, University of California Irvine 8788, Irvine, California, USA; Vanderbilt University Medical Center, Nashville, Tennessee, USA

**Keywords:** sexually transmitted diseases, *Trichophyton*, tinea, case report

## Abstract

**Background:**

*Trichophyton mentagrophytes* complex is emerging as a sexually transmitted infection. Progressive disease, dissemination, or frequent recurrences should raise suspicion for this infection, which often requires prolonged antifungal therapy.

**Case Summary:**

A 37-year-old man and a 35-year-old woman were referred to our infectious diseases clinic for recalcitrant ringworm that had not responded to prior therapy. Fungal cultures from initial visits grew *Trichophyton mentagrophytes* complex, genotypes VII and VIII, respectively. Both patients were successfully treated with a combination of oral and topical azoles.

**Conclusion:**

We report two cases of suspected sexual transmission of *Trichophyton mentagrophytes* complex genotypes VII and VIII in Orange County, California, USA.

## INTRODUCTION

We report the first two documented cases of *T. mentagrophytes* complex genotypes VII (TMVII) and VIII (TMVIII) in Orange County, California, USA.

## CASE PRESENTATION

### Case 1

**Fig 1:**
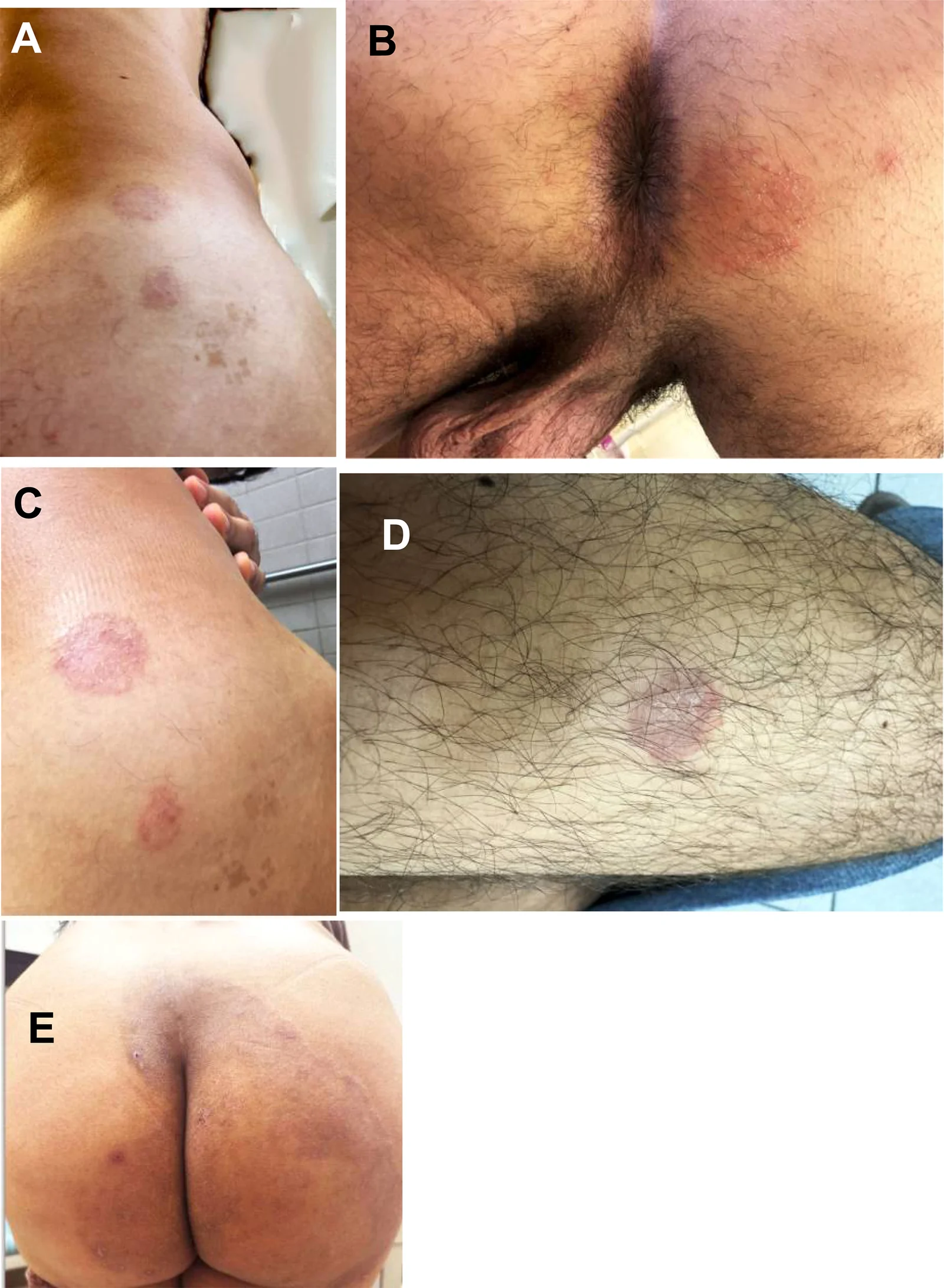
Typical skin lesions due to *Trichophyton mentagrophytes.* (**A–D**) Genotype TMVII: (**A**) hip; (**B**) perianal area; (**C**) shoulder; (**D**) thigh. (**E**) Genotype TMVIII: gluteus.

A 37-year-old man who has sex with men with a history of rectal *Chlamydia trachomatis*, genital herpes, pharyngeal gonorrhea, and syphilis presented to our Infectious Diseases clinic with a recalcitrant pruritic erythematous rash involving the gluteal and perianal regions that had spread to the groin, low back, thighs, shoulder, and forehead. He was taking doxycycline for STI prophylaxis and cabotegravir for HIV pre-exposure prophylaxis. One month prior to presentation, he was evaluated at an urgent care clinic and treated with topical azoles (clotrimazole or ketoconazole) with temporary improvement, followed by progression of the rash. On examination, he had several raised, scaly, erythematous lesions around the anus and right gluteal region with scattered inflamed follicles (Fig. 1A through D). Skin scrapings were obtained for fungal stain and culture, and he was treated with oral and topical azoles (fluconazole and ketoconazole, respectively). His lesions resolved within 4 weeks, with no recurrence at 12 months.

### Case 2

A 35-year-old heterosexual woman with a history of syphilis, HPV, and gonorrhea presented to our Infectious Diseases clinic with a recalcitrant perianal and gluteal rash that developed after a surgical procedure 1 year before her presentation to our clinic. She has been treated with multiple trials of topical glucocorticoids and azoles and ultimately treated with terbinafine (Table 1) without resolution of her rash. On examination, she had numerous erythematous scaly plaques with central clearing on the gluteal regions extending to the perineum and medial thighs (Fig. 1E). Skin scrapings were obtained for fungal studies, and she was prescribed oral and topical azoles (fluconazole and clotrimazole, respectively). After 1 month, the lesions were nearly resolved; however, pruritus persisted in the perineal area. At follow-up, fungal culture and antifungal susceptibility results were available, and treatment was changed to itraconazole, after which symptoms resolved completely.

**TABLE 1:** Clinical presentation, previous treatments, and time to definitive diagnosis of our cases

Case	Age	Gender^*a*^	History of STI	Previous treatments	Months to definitive diagnosis	Definitive diagnosis	Antifungal susceptibility (MIC)	Treatment (po)	Topical treatment
Case 1	37	M	Yes	Topical clotrimazole/ ketoconazole	4	*T. mentagrophyte* genotype VII	Fluconazole 8 µg/mL; itraconazole 0.03 µg/mL, terbinafine <0.0038 µg/mL	Weekly fluconazole 150 mg	Clotrimazole
Case 2	35	W	Yes	Triamcinolone + mupirocin; oral methylprednisolone + topical econazole + flucinomide; terbinafine	13	*T. mentagrophyte* genotype VIII (*T. indotinea*)	Fluconazole 16 µg/mL; itraconazole 0.03 µg/mL, terbinafine 32 µg/mL	Fluconazole 400 mg daily; itraconazole 200 mg BID	Clotrimazole

^*a*^ M, man; W, woman.

### Definitive diagnosis

In both cases, skin scrapings were cultured on inhibitory mold agar (BD BBL) and mycobiotic agar (Hardy Diagnostics) and incubated at 30°C. After 5 days, white, fuzzy colonies were observed and subcultured onto Sabouraud dextrose agar and potato dextrose agar, which showed powdery, fluffy white colonies with tan-to-brown reverse pigmentation. Microscopic examination with lactophenol cotton blue showed septate hyphae with spiral forms, round microconidia, and cigar-shaped septated macroconidia.

Isolates were submitted to the New York State Department of Health Wadsworth Center Mycology Laboratory, Albany (1), for genotyping by internal transcribed spacer (ITS) sequencing and antifungal susceptibility testing. Based on ITS sequencing and BLAST analysis, the organisms were identified as *Trichophyton mentagrophytes* genotypes VII and VIII (*Trichophyton indotineae*), respectively (Table 1).

## DISCUSSION

To our knowledge, these represent the first two cases of suspected sexual transmission of *T. mentagrophytes* complex in Orange County, California. In both patients, the STI history, absence of animal exposure, and perineal-gluteal distribution supported sexual transmission.

*Trichophyton mentagrophytes* complex has emerged as a cause of sexually transmitted fungal skin infections, with the first case reported in 2001 (2) and *tinea genitalis* documented in a heterosexual couple in Denmark in 2009 (3). Though traditionally zoophilic, recent transmission is predominantly human-to-human, mainly through sexual contact among men who have sex with men and sex workers (4–6). More recently, transmission of *Trichophyton mentagrophytes* genotype VII (TMVII), a specific subset, has been reported in several countries, including Italy, France, and Spain (7–10). In 2024, one of the first documented cases in the United States was identified in a patient who had traveled through several European countries and California (11). In that case, a man developed genital lesions after reporting multiple male sexual partners during his travel. In 2024, a separate report from New York City detailed four cases of TMVII infection. All four patients were men who had sex with men and were either on HIV pre-exposure prophylaxis (PrEP) or were receiving antiretroviral therapy for HIV infection (11). Additionally, an observational study from an STI clinic in Spain identified 14 cases of sequence-confirmed TMVII among men who had sex with men (8). Notably, nearly half of the patients in that study required multiple treatment rounds for recurrent lesions.

*T. mentagrophytes* genotype VIII or *T. indotineae* is emerging as a cause of dermatophytosis in recent years. Since 2014, outbreaks have been documented in South Asia (12), where it now causes 78% of dermatophyte infections in India (13). The infection typically begins in the groin and responds poorly to standard therapy, resulting in widespread lesions. Transmission occurs through close contact, including sexual routes (12, 14). *T. indotineae* has recently been identified in Europe, Canada (15), and the United Kingdom (13). Between May 2022 and May 2023, it was confirmed in 11 patients across six New York City medical centers (1, 13, 16).

*T. indotineae* is well-known for its terbinafine resistance, likely a result of the widespread misuse of fixed-dose combination creams containing potent topical corticosteroids (e.g., clobetasol propionate, beclomethasone dipropionate, and mometasone), antifungal agents (e.g., miconazole, clotrimazole, and terbinafine), often antibacterial agents (17), and poor adherence (18). Combination therapy with topical and systemic azoles is typically successful (19).

Both patients experienced delayed diagnosis and received inappropriate topical corticosteroids, which likely contributed to disease progression before referral to our infectious diseases clinic (18). Delays in accurate diagnosis can prolong illness and facilitate spread within the patient and the community (16).

Diagnosis required clinical suspicion and molecular biology techniques, uncommon diagnostic tools for dermatophytosis in primary care. Given its potential for rapid spread, the *T. mentagrophytes* complex should be suspected in recalcitrant rashes. High-risk sexual behavior should prompt further diagnostic testing for proper identification, speciation, and susceptibility testing.

These cases underscore the need for accessible diagnostic tools. The rising incidence of the *T. mentagrophytes* complex warrants increased clinical awareness and patient education.

### Conclusion

Given emerging reports of *T. mentagrophytes* complex infections worldwide, clinicians should maintain a high index of suspicion for dermatophytosis in men who have sex with men, individuals with multiple sexual partners, or those with a history of sexually transmitted infections. Routine fungal culture and ITS sequencing should be considered for recurrent, atypical, or treatment-refractory dermatologic infections. Patient education on the signs and symptoms of resistant fungal infection remains essential for preventing transmission and improving outcomes.

## Data Availability

All supporting data are in this article.

## References

[1] Caplan AS, Todd GC, Zhu Y, Sikora M, Akoh CC, Jakus J, Lipner SR, Graber KB, Acker KP, Morales AE, Rolón RMM, Westblade LF, Fonseca M, Cline A, Gold JAW, Lockhart SR, Smith DJ, Chiller T, Greendyke WG, Manjari SR, Banavali NK, Chaturvedi S. 2024. Clinical course, antifungal susceptibility, and genomic sequencing of trichophyton indotineae. JAMA Dermatol 160:701–709. doi:10.1001/jamadermatol.2024.1126 38748419 PMC11097098

[2] Kupsch C, Czaika V-A, Deutsch C, Gräser Y. 2019. Trichophyton mentagrophytes - a new genotype of zoophilic dermatophyte causes sexually transmitted infections. J Dtsch Dermatol Ges 17:493–501. doi:10.1111/ddg.13776 30775844

[3] Mølenberg D, Deleuran M, Sommerlund M. 2010. Connubial tinea gladiatorum due to Trichophyton mentagrophytes. Mycoses 53:533–534. doi:10.1111/j.1439-0507.2009.01734.x 19682313

[4] Bakare RA, Oni AA, Umar US, Adewole IF, Shokunbi WA, Fayemiwo SA, Fasina NA. 2002. Pattern of sexually transmitted diseases among commercial sex workers (CSWs) in Ibadan, Nigeria. Afr J Med Med Sci 31:243–247. 12751565

[5] Luchsinger I, Bosshard PP, Kasper RS, Reinhardt D, Lautenschlager S. 2015. Tinea genitalis: a new entity of sexually transmitted infection? case series and review of the literature. Sex Transm Infect 91:493–496. doi:10.1136/sextrans-2015-052036 26071391 PMC4680168

[6] Otero L, Palacio V, Vázquez F. 2002. Tinea cruris in female prostitutes. Mycopathologia 153:29–31. doi:10.1023/a:1015257320824 11913763

[7] Jabet Arnaud, Dellière S, Seang S, Chermak A, Schneider L, Chiarabini T, Teboul A, Hickman G, Bozonnat A, Brin C, Favier M, Tamzali Y, Chasset F, Barete S, Hamane S, Benderdouche M, Moreno-Sabater A, Dannaoui E, Hennequin C, Fekkar A, Piarroux R, Normand A-C, Monsel G. 2023. Sexually transmitted trichophyton mentagrophytes genotype VII infection among men who have sex with men. Emerg Infect Dis 29:1411–1414. doi:10.3201/eid2907.230025 37347803 PMC10310379

[8] Descalzo V, Martín MT, Álvarez-López P, García-Pérez JN, Alcázar-Fuoli L, López-Pérez L, Téllez-Velasco D, Carrillo A, Sulleiro E, Falcó V, Arando M. 2025. Trichophyton mentagrophytes genotype VII and sexually transmitted tinea: an observational study in Spain. Mycoses 68:e70049. doi:10.1111/myc.70049 40156344

[9] Bortoluzzi P, Aromolo IF, Derlino F, Marzano AV, Perego G. 2025. Tinea barbae caused by T. mentagrophytes genotype VII, an emerging sexually transmitted infection among Men who have sex with men: a report from Milan, Italy. J Eur Acad Dermatol Venereol 39:e187–e190. doi:10.1111/jdv.20395 39587982

[10] Jabet A, Bérot V, Chiarabini T, Dellière S, Bosshard PP, Siguier M, Tubiana R, Favier M, Canestri A, Makhloufi S, et al.. 2025. Trichophyton mentagrophytes ITS genotype VII infections among men who have sex with men in France: an ongoing phenomenon. J Eur Acad Dermatol Venereol 39:407–415. doi:10.1111/jdv.20439 39587983 PMC11760687

[11] Caplan AS, Sikora M, Strome A, Akoh CC, Otto C, Chaturvedi S, Zampella JG. 2024. Potential sexual transmission of tinea pubogenitalis from TMVII. JAMA Dermatol 160:783–785. doi:10.1001/jamadermatol.2024.1430 38837127

[12] Abdolrasouli A, Barton RC, Borman AM. 2025. Spread of antifungal-resistant trichophyton indotineae, United Kingdom, 2017-2024. Emerg Infect Dis 31:192–194. doi:10.3201/eid3101.240923 39714510 PMC11682803

[13] Marbaniang YV, Leto D, Almohri H, Hasan MR. 2025. Treatment and diagnostic challenges associated with the novel and rapidly emerging antifungal-resistant dermatophyte, *Trichophyton indotineae* J Clin Microbiol 63:e0140724. doi:10.1128/jcm.01407-24 40497720 PMC12153305

[14] Spivack S, Gold JAW, Lockhart SR, Anand P, Quilter LAS, Smith DJ, Bowen B, Gould JM, Eltokhy A, Gamal A, Retuerto M, McCormick TS, Ghannoum MA. 2024. Potential sexual transmission of antifungal-resistant trichophyton indotineae. Emerg Infect Dis 30:807–809. doi:10.3201/eid3004.240115 38437706 PMC10977831

[15] Smith A, Wong-O’Brien B, Lieberman JA, Cookson BT, Grinager E, Truong TT. 2024. The Brief Case: a case of tinea corporis caused by drug-resistant *Trichophyton indotineae* identified by broad-range fungal DNA sequencing. J Clin Microbiol 62:e0023424. doi:10.1128/jcm.00234-24 39140757 PMC11323470

[16] Caplan AS, Chaturvedi S, Zhu Y, Todd GC, Yin L, Lopez A, Travis L, Smith DJ, Chiller T, Lockhart SR, et al.. 2021. Notes from the field: first reported U.S. Cases of tinea caused by trichophyton indotineae- New York City, December 2021-March231 2023. MMWR Morb Mortal Wkly Rep 72:536–537. doi:10.15585/mmwr.mm7219a4 PMC10208369 37167192

[17] Dos Santos AR, Uhrlaß S, Nenoff P, Gold JAW, Bhuiyan MSI, Goturu S, Gade L, Bagal UR, Peterson JG, Wiederhold NP, Lockhart SR, Järv H, Hameed AF, Szepietowski J, Vasani R, Singal A, Parnell L, Schwem B, Chiller T, Litvintseva AP, Chow NA, Verma SB. 2025. Global emergence of antifungal-resistant dermatophytosis caused by *Trichophyton indotineae* (Formerly *T. mentagrophytes* ITS Genotype VIII): a genomic investigation involving 14 Countries. Mycoses 68:e70101. doi:10.1111/myc.70101 40808374 PMC12393040

[18] Bui TS, Chan JB, Katz KA. 2024. Extensive multidrug-resistant dermatophytosis from Trichophyton indotineae. Cutis 113:E20–E23. doi:10.12788/cutis.1050 39082990

[19] Sonego B, Corio A, Mazzoletti V, Zerbato V, Benini A, di Meo N, Zalaudek I, Stinco G, Errichetti E, Zelin E. 2024. *Trichophyton indotineae*, an emerging drug-resistant dermatophyte: a review of the treatment options. J Clin Med 13:3558. doi:10.3390/jcm13123558 38930086 PMC11204959

